# Plant growth responses to elevated atmospheric CO_2_ are increased by phosphorus sufficiency but not by arbuscular mycorrhizas

**DOI:** 10.1093/jxb/erw383

**Published:** 2016-10-17

**Authors:** Iver Jakobsen, Sally E. Smith, F. Andrew Smith, Stephanie J. Watts-Williams, Signe S. Clausen, Mette Grønlund

**Affiliations:** ^1^Department of Chemical and Biochemical Engineering, Technical University of Denmark, DK-2800 Kgs. Lyngby, Denmark; ^2^ Present address: Department of Plant and Environmental Sciences, Faculty of Science, University of Copenhagen, DK-1871, Thorvaldsensvej 40, Frederiksberg C, Denmark.; ^3^ Present address: Soils Group, School of Agriculture, Food and Wine, Waite Campus, The University of Adelaide, SA 5005, Australia.; ^4^ Present address: Boyce Thompson Institute, Tower Rd, Ithaca, NY 14853, USA.

**Keywords:** Arbuscular mycorrhizal symbiosis, *Brachypodium distachyon*, elevated atmospheric CO_2_, gene expression, *Medicago truncatula*, phosphate transporters, plant growth, plant phosphorus uptake, soil phosphorus.

## Abstract

Capturing the full growth potential in crops under future elevated CO_2_ (eCO_2_) concentrations would be facilitated by improved understanding of eCO_2_ effects on uptake and use of mineral nutrients. This study investigates interactions of eCO_2_, soil phosphorus (P), and arbuscular mycorrhizal (AM) symbiosis in *Medicago truncatula* and *Brachypodium distachyon* grown under the same conditions. The focus was on eCO_2_ effects on vegetative growth, efficiency in acquisition and use of P, and expression of phosphate transporter (PT) genes. Growth responses to eCO_2_ were positive at P sufficiency, but under low-P conditions they ranged from non-significant in *M. truncatula* to highly significant in *B. distachyon*. Growth of *M. truncatula* was increased by AM at low P conditions at both CO_2_ levels and eCO_2_×AM interactions were sparse. Elevated CO_2_ had small effects on P acquisition, but enhanced conversion of tissue P into biomass. Expression of PT genes was influenced by eCO_2_, but effects were inconsistent across genes and species. The ability of eCO_2_ to partly mitigate P limitation-induced growth reductions in *B. distachyon* was associated with enhanced P use efficiency, and requirements for P fertilizers may not increase in such species in future CO_2_-rich climates.

## Highlight

Experiments combining elevated CO_2_ (eCO_2_), mycorrhizas and soil P level revealed that eCO_2_ increases the P use efficiency in *Medicago truncatula* and *Brachypodium distachyon* but not their mycorrhiza-mediated P uptake.

## Introduction

Dramatic increases in atmospheric concentrations of carbon dioxide (CO_2_) since pre-industrial times are predicted to produce CO_2_ levels of ~500 to ~900 ppm by the end of this century, according to different climate scenarios (IPCC, 2013). Elevated CO_2_ concentrations (eCO_2_) are expected to increase growth of C_3_ plants primarily because the current CO_2_ concentration is suboptimal for the Rubisco enzyme that catalyzes carbon fixation; in particular, eCO_2_ will competitively inhibit the oxygenation reaction and so reduce CO_2_ loss and energy costs associated with photorespiration.

Other factors in the growth environment such as soil phosphorus (P) levels will influence the magnitude of the ‘carbon fertilizer’ effects on future crop productivity (Cavagnaro *et al.*, 2011; Pandey *et al.*, 2015*b*) and many soils are already characterized by decreasing P availability (Obersteiner *et al.*, 2013). Global abundance of such soils may further increase as rock P is non-renewable on a human time scale (Scholz and Wellmer, 2013), or because P fertilizer becomes prohibitively expensive for farmers, especially in developing countries. It is therefore important to understand if or how the expected eCO_2_ effects on crop yields can be realized under P-limiting conditions (Pandey *et al.*, 2015*a*). Possible requirements for higher inputs of P fertilizers under eCO_2_ conditions could even accelerate the depletion of rock P reserves.

In general, the efficiency of plant acquisition and utilization of soil P should be maximized to sustain food production in low-P soils (López-Arredondo *et al.*, 2014) and attempts to improve production need to consider interactions with eCO_2_. Plant P acquisition is enhanced by extensive development of roots and is therefore determined by the C status of plants; on the other hand, the P status influences plant photosynthesis and growth rate, leading to multiple C–P interactions. The physiological background for the C–P trade balance as influenced by eCO_2_ and low P conditions are appropriately investigated in experiments under controlled conditions to minimize possible masking of C–P trading by non-nutritional influences that are common under field conditions. Previous studies on eCO_2_×P interactions have shown that eCO_2_ can increase growth of C_3_ grasses even in low-P soil (Newbery *et al.*, 1995; Imai and Adachi, 1996; Newbery and Wolfenden, 1996; Pandey *et al.*, 2015*a*), whereas the response in legumes (also C3) is usually limited under low-P conditions (Stocklin and Körner, 1999; Edwards *et al.*, 2005; Jin *et al.*, 2012; Lam *et al.*, 2012; Singh *et al.*, 2014). Such differences between functional plant groups are influenced by patterns of C partitioning and by efficiencies in P acquisition by their root systems (Jin *et al.*, 2015). Plant responses to eCO_2_ are likely to be modulated by their mutualistic root symbionts, and there is a body of evidence showing that eCO_2_ increases nitrogen fixation in legumes (Rogers *et al.*, 2009). It is also highly relevant to ask whether eCO_2_ will amplify the development and function of the arbuscular mycorrhizal (AM) symbiosis that delivers soil P to most land plants in return for photosynthate, resulting in increased growth responses to eCO_2_ via positive feedback. A possible C limitation of AM development both in the soil and within the roots might be mitigated at eCO_2_ and lead to increased mycorrhiza formation. This may, in turn, result in an increased mycorrhizal P uptake and facilitate an eCO_2_ growth response in (e.g.) legumes grown at low P.

Two meta-analyses have found that the abundance of AM colonization increases under eCO_2_ (Treseder, 2004; Alberton *et al.*, 2005), but data are variable and can be positive or neutral (Gavito *et al.*, 2002, 2003; Hartwig *et al.*, 2002; Lukac *et al.*, 2003; Gamper *et al.*, 2004; Cavagnaro *et al.*, 2007). The eCO_2_ effect on growth of AM plants will depend on the AM C–P trade balance. Growth responses to eCO_2_ were reported to be similar for AM-colonized and non-colonized *Pisum sativum* and *Trifolium repens* (Jongen *et al.*, 1996; Gavito *et al.*, 2000, 2002, 2003; i.e. there were no CO_2_×AM interactions), but AM-amplified growth responses to eCO_2_ have also been reported (Rouhier and Read, 1998; Hartwig *et al.*, 2002). Even when eCO_2_ has no net effect on growth of AM plants, there may still be a concealed physiological effect. That is, P uptake in AM plants is the combined contribution of the direct root pathway and of the AM pathway, and the latter can be highly functional even when overall growth and P uptake are similar in AM and non-mycorrhizal (NM) plants, indicating reductions in the activity of the direct pathway (Smith *et al.*, 2004; Grace *et al.*, 2009; Nagy *et al.*, 2009). The function of the AM pathway can only be quantitatively assessed by using radioactive phosphate (^33^P or ^32^P; Pearson and Jakobsen, 1993), but the potential function can be evaluated in studies of expression of phosphate (Pi) transporter (PT) genes and combined approaches are becoming increasingly common (see Smith *et al.*, 2011). The two P uptake pathways involve a number of PT proteins, some of which are specific to or induced by AM fungi (Javot *et al.*, 2007*b*; Smith *et al.*, 2011). Importantly for this investigation, the PT genes expressed in roots have been identified in both *Brachypodium distachyon* (Hong *et al.*, 2012) and *Medicago truncatula* (Liu *et al.*, 2008). In *M. truncatula*, roles in direct and AM pathways have been identified, but roles of individual genes are less clear in *B. distachyon*. Few studies have analyzed the effects of eCO_2_ on PT gene expression, and these have been in the non-AM plant *Arabidopsis thaliana* (Niu *et al.*, 2013; Pandey *et al.*, 2015*b*).

As soil P availability is a major determinant for growth responses to AM symbiosis, it is striking that this factor has been considered in only a few studies concerning eCO_2_×AM interactions, which have included only two experimentally imposed P levels (Syvertsen and Graham, 1999; Johnson *et al.*, 2005). In the first case, the eCO_2_ response in Citrus was amplified by AM symbiosis under P limitation, while such an effect was not observed in the other study that included a range of plant species. There is an obvious need for more studies on eCO_2_×AM interactions, including not only a wider range of soil P availability but also different plant species (Syvertsen and Graham, 1999; Johnson *et al.*, 2005; Cavagnaro *et al.*, 2011).

The aim of this work was to study how soil P level and AM symbiosis influence eCO_2_ effects on growth of two plant species differing in responses to soil P limitation and to AM colonization: *Medicago truncatula* Gaertn. (barrel medic) and *Brachypodium distachyon* (L.) P. Beauv. *Medicago truncatula* has been well studied; it generally shows positive growth and P responses to AM colonization and the contribution of the AM P uptake pathway has been tracked with ^32/33^P (see for example Smith *et al.*, 2004; Facelli *et al.*, 2014; Watts-Williams *et al.*, 2015). It has rather poor P uptake efficiency when non-mycorrhizal (NM). *Brachypodium distachyon* has (to our knowledge) been subject to only one investigation involving AM symbioses (Hong *et al.*, 2012). Responses in low-P soil based on fresh shoot weight or P content varied with the symbiotic AM fungus and were in some cases neutral or negative. It was expected that this species would show quite high P uptake efficiency, regardless of mycorrhizal status.

Thus, the following hypotheses were tested: (1) growth responses to eCO_2_ under low-P conditions depend on the efficiency of P acquisition and use in the plant species when AM or NM; (2) growth responses to eCO_2_ will increase with increasing soil P levels; and (3) AM functioning is little affected by eCO_2_.

## Materials and methods

A pot experiment was carried out at both ambient (aCO_2_ = 400 ppm) and elevated (eCO_2_ = 900 ppm) atmospheric CO_2_ concentrations, with *M. truncatula* cv. Jemalong A17 and *B. distachyon* line 21–3 growing in soil supplied with 0, 10, 20, 40, or 80 mg KH_2_PO_4_-P kg^−1^. Plants were inoculated with an AM fungus or not.

### Experimental set-up

Seeds of *M. truncatula* were scarified in concentrated H_2_SO_4_ for 8 min, rinsed in sterile water, surface-sterilized with 2% NaHClO_3_ for 5 min, rinsed in sterile water and pre-germinated on water-agar (0.8%) plates in the dark at 4 ^o^C (5 d) and at 22 ^o^C (2 d). Seeds of *B. distachyon* were surface-sterilized and pre-germinated in the same way.

The experimental soil was a semi-sterile (15 kGy, 10 MeV electron beam) 1:1 (w:w) mixture of a sandy loam (10% clay, 12% silt, 46% fine sand, and 30% coarse sand) and quartz sand, which was supplemented and thoroughly mixed with basal nutrients (Merrild *et al.*, 2013). The five P treatments are referred to as 0P, 10P, 20P, 40P, and 80P and resulted in the following levels of 0.5 M NaHCO_3_-extractable P (Olsen *et al.*, 1954): 4.3, 7.8, 11.2, 19.8, and 39.6 mg P kg^−1^ soil.

The pots held 1430 g soil, of which 50 g was mixed with 262 kBq of carrier-free H_3_^33^PO_4_. This labelled soil was contained in a 40-mm diameter plastic cylinder capped with 25-µm nylon mesh at both ends, and this compartment for ingrowth of AM fungal hyphae (hyphal compartment: HC) was placed at 10 cm depth in all pots (see Smith *et al.*, 2003, which shows a diagram of the compartmented pot). Mycorrhizal pots had a mixture of 1000 g soil and 100 g inoculum of *Rhizophagus irregularis* (Blaszk., Wubet, Renker & Buscot) C.Walker & A.Schüßler 2010 (previously named *Glomus intraradices*) culture BEG87 sandwiched between the bottom (200 g) and top (80 g) layers of non-inoculated soil. The AM fungal inoculum was a mixture of dry soil, spores, and root fragments of *Trifolium subterraneum* L. pot cultures. All NM and AM pots received 15 ml inoculum leachate that was prepared by wet-sieving 1 l aqueous suspension of 100 g inoculum through two layers of 25-µm nylon mesh. Each of the 15 treatments for each species had three replicates.

The soil in the pots was watered to 60% of the water-holding capacity and pots were placed in two separate walk-in growth rooms set at 400 and 900 ppm CO_2_. Two or three pre-germinated seeds were sown in each pot and emerged seedlings were thinned to one per pot. Plants were maintained at a 16/8 h light/dark cycle at 20/15 °C, respectively. Fluorescent daylight lamps (Osram GmbH, Munich, Germany) provided 500 μmol m^–2^ s^–1^ photosynthetically active radiation (PAR; 400–700 nm). As plants grew bigger, pots were watered to 70% of water-holding capacity and fertilized twice with NH_4_NO_3_, resulting in a total supply of 112 mg N per pot. To avoid chamber-specific bias in the experiment, pots and their corresponding CO_2_ treatment were relocated between the two climate chambers every week.

### Harvesting and physiological analysis

Plants were harvested 35 d after sowing (about half the life-cycles of the two species); shoots were dried at 70 °C for 48 h and dry weights recorded. Harvest time was chosen to take into account the half-life of ^33^P (25.4 d) and the influence of P supply on the specific activity, and hence detectability, of ^33^P transferred to the plants. Roots were washed, blotted, and weighed, and a weighed subsample of 500–700 mg was stored in 50% ethanol for determination of AM colonization. Another ~500 mg subsample of root material was flash-frozen in liquid N_2_, crushed, and kept at −80 °C for RNA isolation. The remaining root tissue was dried at 70 °C for 48 h and dry weights were determined. Growth responses to eCO_2_ (% eCO_2_ response) and to AM inoculation (% AM response) were calculated from shoot dry weights as follows: % eCO_2_ = 100×(eCO_2_ – mean aCO_2_)/mean aCO_2_, and % AM = 100×(AM – mean NM)/mean NM. Dried shoot and root samples were digested in a 4:1 mixture (v:v) of 65% nitric and 70% perchloric acids, and total P was determined by the molybdate blue method using AutoAnalyzer 3 (Bran + Luebbe, Norderstedt, Germany). The ^33^P in shoot and root tissue was determined in the same digests in a Packard TR 1900 liquid scintillation counter (PerkinElmer, Waltham, MA).

Root samples were cleared in 10% KOH and stained with trypan blue (Kormanik and McGraw, 1982), and were then assessed for AM-colonized root length (Newman, 1966). Quantification of hyphae in the root-free HC soil was investigated for *M. truncatula* by measuring the length of hyphae collected on membrane filters (Jakobsen *et al.*, 1992). After correction for isotopic decay, uptake of ^33^P from the small HCs (in which the soil specific activity was measured and varied between P treatments) was extrapolated to uptake from the whole pot as described previously (Smith *et al.*, 2003) to give % of total plant P uptake by AM fungal hyphae: 100 × (SA^33^P plant/SA^33^P HC) × (P in pot/P in HC), where SA is specific activity and P is bicarbonate-extractable P. The calculations did not take into account the possibility of different hyphal length densities (HLDs) in the main pot and HC (Smith *et al.*, 2004) as HLDs were only measured in the HCs.

### RNA isolation and real-time qPCR analysis

Total RNA was extracted from ~70 mg of root samples of both species, using miRNeasy Mini Kit (Qiagen Hilden, Germany) with on-column DNase treatment following the manufacturer’s protocol. RNA concentration was measured on a Nanodrop ND-1000 Spectrophotometer (Saveen 1 Werner, Malmö, Sweden). cDNA was synthesized from 200 ng of total RNA using dNTP Mix (Qiagen) and Expand Reverse Transcriptase (Roche) including Protector RNase inhibitor (Roche).

The real-time primers (Eurofins MWG operon, Germany) were: *MtEF1α*, *MtPT1*, *MtPT3*, and *MtPT5* (Liu *et al.*, 2008); *MtPT4* (Javot *et al.*, 2007*a*), and *BdUBC18* (Hong *et al.*, 2008). Primers for *BdPT4*, *BdPT8*, and *BdPT7* are given in Supplementary Table SI at *JXB* online. Gene expression analysis was carried out on three replicate plants from each treatment, with technical duplicates. Real-time PCR analysis was performed using the Rotor Gene 2000 Real Time Cycler (Qiagen). Each 20 μl of PCR reaction contained 8 μl of a 1/8 dilution of RT reaction (see above), and 12 μl of SYBR Green Master Mix (Fermentas, Thermo Scientific), which included 500 nM of each primer. Samples were heated to 95 **°**C for 10 min, followed by 40 cycles of 15 s at 95 **°**C and 1 min at 60 **°**C. After each PCR reaction, the specificity of the amplification was verified by running a melt-curve analysis. The Rotor Gene 2000 software calculated relative amounts of RNA based on PCR cycle threshold values obtained from a dilution series from 1/4 to 1/160 (each step was a 1:3 dilution in H_2_O) of a standard RT sample from an AM or NM plant (depending on the primer of interest). Data were normalized to *MtEF1α* mRNA levels.

### Statistics

All data were assessed for normality using the Shapiro–Wilk test and by viewing QQ-plots. Any data that appeared non-normal were square-root or log transformed so that they conformed to the assumption of normality before further statistical analysis. All response variable data (except for gene expression, % eCO_2_ response and % AM response) were analyzed by multi-factor analyses of variance (ANOVA). Factors in the three-way analyses were: CO_2_ level, soil P level, and arbuscular mycorrhiza. Root colonization was analyzed by two-way ANOVA (CO_2_ level and soil P level) after removing the nil-values for NM plants. Gene expression data were split between the AM and non-mycorrhizal (NM) treatments and also analyzed by two-way ANOVA (CO_2_ level and soil P level). For % eCO_2_ response, the factors in the two-way ANOVA were arbuscular mycorrhiza and soil P level, while for % AM response the factors were CO_2_ level and soil P level. Where significant (*P*<0.05) interactions or main effects were found, comparisons were made using Tukey’s honestly significant difference (HSD). Linear or polynomial regression analyses were performed in Microsoft Excel (version 14.5.1) to determine the relationship between shoot dry weights and shoot P contents (the P use efficiency, PUE) at each CO_2_ level, respectively. All other statistical analyses were performed with JMP Pro 12.0.1 (SAS Institute Inc.).

## Results

Table 1 shows probabilities of significance for the main treatment effects and treatment interactions derived from ANOVA.

**Table 1.** Probabilities of significance for main treatment effects and treatment interactions of the variables measured in M. truncatula and B. distachyon as derived from three-way ANOVA. Gene expression data were analyzed separately for AM and NM plants by two-way ANOVA. Mycorrhizal and CO_2_ growth responses (% AM response, % eCO_2_ response) were also analyzed by two-way ANOVA.

Variable	AM	CO_2_	P level	AM×CO_2_	AM×P	CO_2_×P	AM×CO_2_×P
*M. truncatula*							
Shoot DW	<0.0001	<0.0001	<.0001	ns*	<0.0001	0.001	ns
Shoot P conc	<0.0001	<0.0001	<0.0001	0.042	<0.0001	ns	ns
Shoot P cont	<0.0001	0.0025	<0.0001	0.0044	<0.0001	ns	ns
Root colonization		ns	0.0002			ns	
Root DW	<0.0001	<0.0001	<0.0001	ns	<0.0001	ns	ns
Root length	ns	0.041	<0.0001	ns	0.007	ns	ns
RL-spec P uptake	<0.0001	ns	<0.0001	ns	0.001	ns	ns
AM P uptake	<0.0001	ns	0.0123	ns	<0.0001	ns	ns
% AM response		0.001	<0.0001			0.0273	
% eCO_2_ response	0.0129		ns		ns		
Expression of PT genes**							
*MtPT1* NM		ns (0.056)	0.002			ns	
*MtPT3* NM		0.038	ns			ns (0.096)	
*MtPT5* NM		<0.0001	ns			ns	
*MtPT4* AM***		0.001	0.004			ns	
*MtPT1* AM		0.001	0.001			ns	
*MtPT3* AM		<0.0001	0.003			ns	
*MtPT5* AM		0.004	ns			ns	
*B. distachyon*							
Shoot DW	ns	<0.0001	<0.0001	ns	ns	0.007	ns (0.087)
Shoot P conc	ns	ns	<0.0001	ns	ns (0.057)	ns	ns
Shoot P cont	ns	<0.0001	<0.0001	ns	0.0193	ns	0.0287
Root colonization		0.0077	<0.0001	0.004	<0.0001	ns	ns
RL-spec P uptake	ns	ns	<0.0001	ns	ns	ns	ns
Root DW	0.0322	<0.0001	<0.0001	ns	ns	0.0009	ns
Root length	ns	<0.0001	0.004	ns	ns	ns	ns
AM P uptake	<0.0001	ns	0.0093	ns	<0.0001	ns	ns
% AM response		ns	0.042			ns (0.052)	
% eCO_2_ response	ns		0.002		0.011		
Expression of PT genes**							
*BdPT4* NM		ns	0.0003			ns	
*BdPT8* NM		0.009	<0.0001			ns	
*BdPT7* AM***		ns (0.066)	0.0003			ns	
*BdPT4* AM		0.021	ns			ns	
*BdPT8* AM		ns	0.0002			ns	

* ns, not significant

** no AM component in ANOVA since NM and AM plants were analysed separately

*** *MtPT4* and *BdPT7* are not expressed in NM plants

### Effects of P fertilization and elevated CO_2_ on plant growth and root colonization by AM fungi

**Fig. 1.**
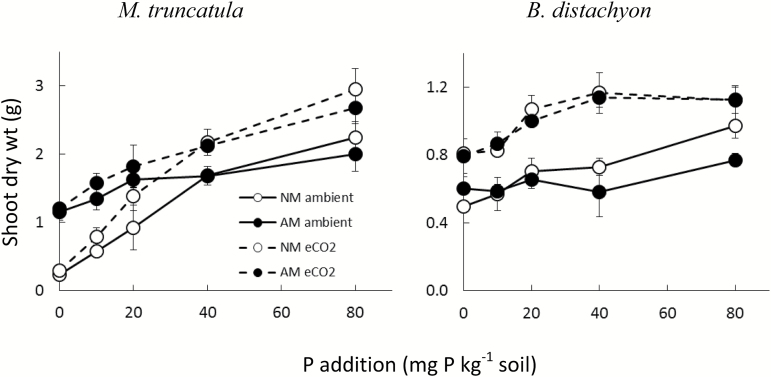
Shoot dry weights of *M. truncatula* and *B. distachyon* grown at aCO_2_ (solid lines) and eCO_2_ (dashed lines), in the presence or absence of AM colonization (AM or NM: filled or open symbols) and at different soil P levels. Data points are means ±SEM with *n*=3.

Growth of both plant species increased significantly with increasing P fertilization, but the effect was much stronger for *M. truncatula* than for *B. distachyon*, with shoot dry weight in the legume failing to reach a plateau (Fig. 1). Furthermore, interactions between soil P supply and inoculation by AM fungi differed between species (Table 1), as discussed below.

Growth responses to eCO_2_ differed markedly between the legume and the grass. In *M. truncatula*, growth was severely P-limited in the 0P–20P range and was only increased by eCO_2_ (% eCO_2_ response) at the two highest P levels, as reflected by the significant CO_2_×P interaction (Fig. 1, Tables 1 and 2). The eCO_2_ response was similar in AM and NM plants although the P-limited growth was partly mitigated by AM colonization (Tables 1 and 2, Fig. 1). In contrast, shoot DW of *B. distachyon* was significantly higher at eCO_2_ than at aCO_2_ at all P levels except the highest (80P). Further, at eCO_2_, dry weight accumulation reached a plateau at 40P in *B. distachyon*, while no such plateau was observed in the plants grown at aCO_2_.

Mycorrhizal growth response (% AM response, Table 2) was strongest for *M. truncatula*, due to the suppression of growth at 0P when non-mycorrhizal. Growth of *M. truncatula* responded significantly to AM development in the 0P to 20P range and responses were lowest at eCO_2_ (Fig. 1, Tables 1 and 2). With both CO_2_ treatments, % AM response declined with increasing soil P level in accordance with the significant AM×P interaction (Table 1). Colonization by AM fungi had no significant effects on growth of *B. distachyon* and responses to the addition of P were matched between the AM and NM plants at eCO_2_ (Tables 1 and 2, Fig. 1). At aCO_2_ the data trended towards a positive, but then negative % AM response at 0P and 80P, respectively (Tables 1 and 2). The effect of CO_2_, AM colonization, and soil P treatments on root dry weights closely reflected the shoot growth (Supplementary Fig. S1).

**Table 2.** Relative shoot growth responses to elevated CO_2_ [= 100×(eCO_2_ – mean aCO_2_)/mean aCO_2_] and to AM inoculation [= 100×(AM – mean NM)/mean NM] in *M. truncatula* and *B. distachyon* grown at different soil P supplies.

P supply (mg kg^−1^)	% CO_2_ response				% AM response			
	NM		AM		aCO_2_		eCO_2_	
	Mean	SEM	Mean	SEM	Mean	SEM	Mean	SEM
			*M. truncatula*					
0	25.0	13.4	4.3	2.5	381.9	32.0	302.2	9.7
10	36.2	12.1	17.4	4.8	131.0	15.6	99.2	8.1
20	49.6	14.7	12.1	8.5	75.5	6.8	31.5	9.9
40	28.9	4.9	27.0	1.5	−0.4	2.8	−1.8	1.2
80	31.1	8.5	33.4	7.0	−11.0	5.4	−9.4	4.8
			*B. distachyon*					
0	63.3	5.1	31.8	13.8	21.1	11.8	−2.2	10.3
10	45.1	3.3	47.5	9.7	3.1	5.5	4.8	6.9
20	52.0	9.1	52.2	4.7	−6.7	6.3	−6.6	2.9
40	59.7	13.5	95.3	8.3	−20.1	11.8	−2.3	4.2
80	15.6	4.6	46.5	8.5	−20.8	3.2	0.3	5.8

**Fig. 2.**
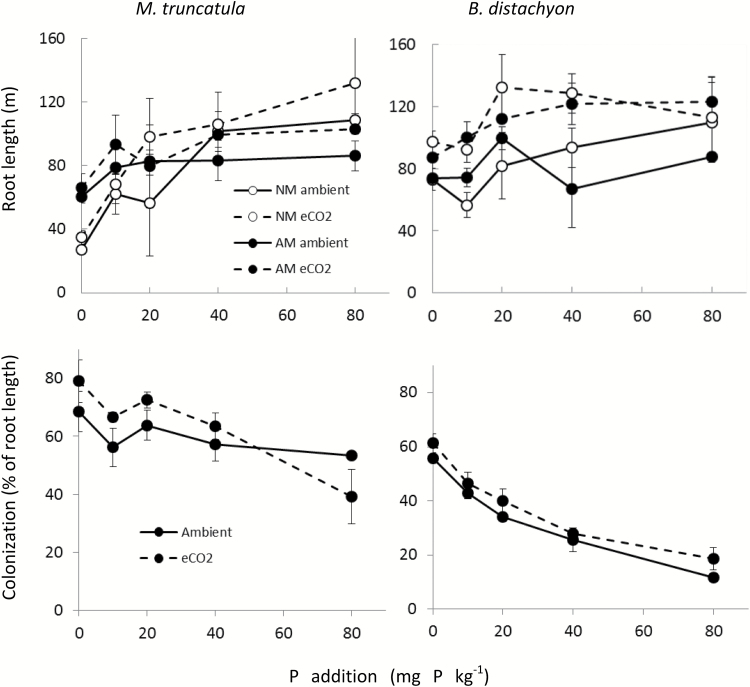
Total root length of *M. truncatula* and *B. distachyon* grown at aCO_2_ (solid lines) and eCO_2_ (dashed lines), in the presence or absence of AM colonization (AM or NM: filled or open symbols) and at different soil P levels. Percentage of root length colonized is shown in the lower panel for AM plants of the two species grown at aCO_2_ and eCO_2_ and different soil P levels. Data points are means ±SEM with *n*=3.

Elevated CO_2_ also resulted in increased root length in both *M. truncatula* and *B. distachyon* (Fig. 2). Colonization by AM fungi did not affect root length in *B. distachyon*, but in *M. truncatula* root length was increased and decreased by AM symbiosis at low-P and high-P conditions, respectively. This modification by AM colonization was associated with a lower specific root length (m g^−1^ DW) in AM plants, in particular in the 0P to the 20P range (data not shown). Roots of all inoculated plants were colonized by AM fungi and non-inoculated plants remained non-colonized. Percentage of root length colonized at 0P was higher than 55% in both species and decreased significantly with increasing P application. This decrease was largest in *B. distachyon* (from ~60% at 0P to 20% at 80P) (Fig. 2). The percentage of root length colonized was not strongly affected by eCO_2_ except for moderate but significant increases in *B. distachyon* (Fig. 2). However, because eCO_2_ increased total root length, the absolute length of colonized roots was increased by approx. 10% in *M. truncatula* and by as much as 50% in *B. distachyon* grown at P40 (data not shown).

### Effects of elevated CO_2_ on shoot P concentrations and P use efficiency

**Fig. 3.**
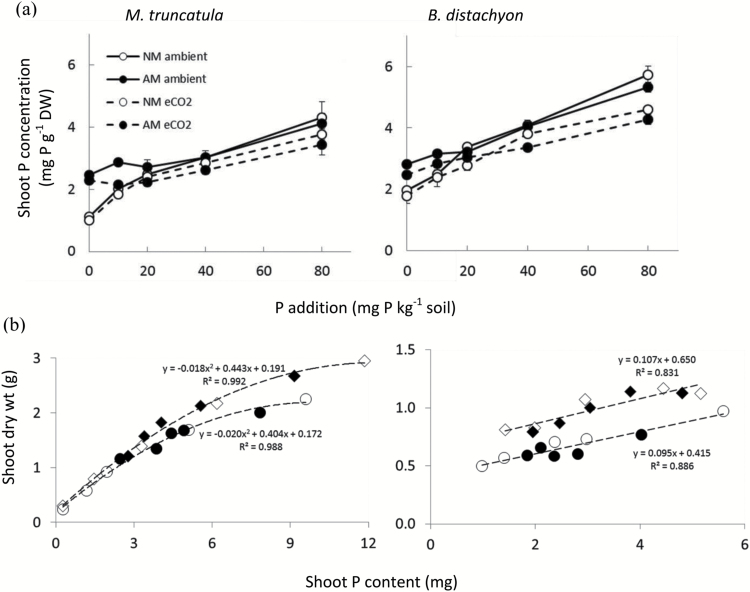
Phosphorus concentrations (a) and dry weight vs. P content relationships (b) for shoots of *M. truncatula* and *B. distachyon* grown at aCO_2_ (solid lines) and eCO_2_ (dashed lines) (a), in the presence or absence of AM colonization (AM or NM: filled or open symbols) and at different soil P levels. In (b), aCO_2_ and eCO_2_ treatments are denoted by circles and diamonds, respectively, and P use efficiency is derived from the *y*/*x*-axis ratios. Data points are means (*n*=3) and bars in (a) are ±SEM.

Shoot tissue P content in both species increased significantly over the range of P application, as did shoot P concentrations (Table 1, Supplementary Fig. S2, Fig. 3a). Exposure to eCO_2_ resulted in decreased shoot P concentrations in *M. truncatula* at higher soil P levels. In *B. distachyon* eCO_2_ did not alter tissue P concentrations significantly. Root colonization by AM fungi increased P concentrations in *M. truncatula* at 0P and 10P (Fig. 3a). This reflected a general shift from a positive AM effect at the low P levels towards a neutral or negative effect at the highest P levels. Shoot P concentrations in *M. truncatula* were similar in AM-colonized plants grown at 0P and in NM plants grown at 20P (Fig. 3a).

The P use efficiency (PUE), being the reciprocal of shoot P concentration, is derived from the *y*/*x*-axis ratios in Fig. 3b, which shows the relationship between shoot dry weight and shoot P content. This relationship, which facilitates the analysis of AM or CO_2_ treatment effects on PUE in plants having similar P contents, was curvilinear in *M. truncatula*, where PUE was increased by eCO_2_ when P uptake was beyond a certain threshold (about 3 mg P). In *B. distachyon*, the relationship was linear and PUE was increased by eCO_2_ over the full range of shoot P contents studied (Fig. 3b). In contrast, PUE seemed to be unaffected by AM colonization when comparing plants of similar shoot P content in each species.

### No effects of elevated CO_2_ on root length-specific P uptake and AM contribution to P uptake

**Fig. 4.**
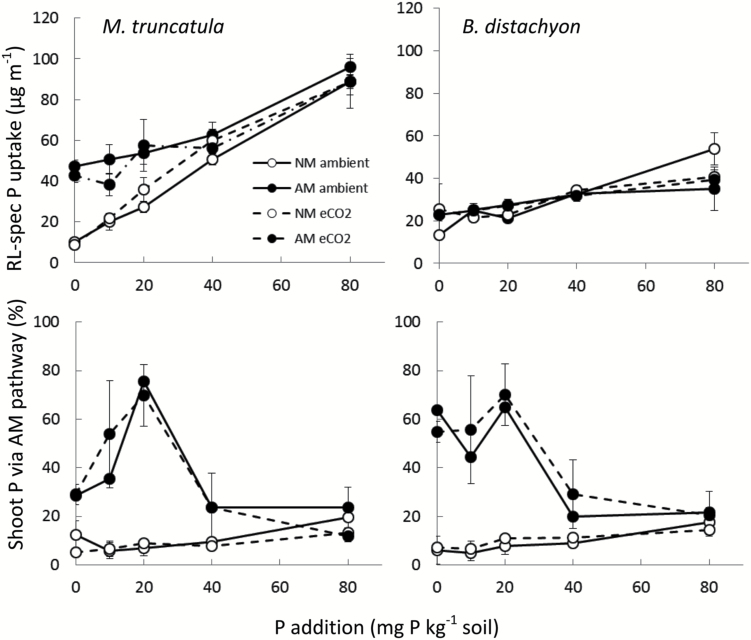
Root length (RL)-specific P uptake in *M. truncatula* and *B. distachyon* grown at aCO_2_ (solid lines) and eCO_2_ (dashed lines), in the presence or absence of AM colonization (AM or NM: filled or open symbols) and at different soil P levels. Shoot P uptake via the AM pathway is shown in the lower panel. Data points are means ±SEM with *n*=3.

Uptake of P per unit root length (root length-specific: RL-spec) was not significantly affected by the CO_2_ level, but increased in response to P addition in both species and in response to AM root colonization in *M. truncatula* (up to 20P only), but not in *B. distachyon* (Table 1, Fig. 4). The effect of P and AM colonization interacted significantly in *M. truncatula*, such that RL-spec P uptake increased more steeply with increasing P addition in NM than in AM plants.

The contribution of the AM pathway to uptake was estimated from ^33^P uptake via hyphae accessing the small hyphal compartments (HCs) containing ^33^P-labelled soil. Irrespective of the different AM growth response observed in the two species, the calculation showed that they both received 65–75% of their shoot P uptake via the AM pathway at 20P, and that this was not affected by CO_2_ level (Fig. 4). The activity of the AM pathway decreased markedly between 20P and 40P and appeared to be non-operational at 80P in both species. In AM *M. truncatula*, the calculated uptake of ^33^P was clearly lower at 0P and 10P than at 20P. However, there was a linear correlation between hyphal length densities in the HC soil and ^33^P uptake from the same soil (Supplementary Fig. S3). The uptake of ^33^P by NM plants was very low with low soil P and increased with increasing soil P level (Fig. 4). This uptake was probably caused by the combined effect of root hairs penetrating the mesh and diffusion of ^33^P in the opposite direction.

### Effects of elevated CO_2_ on expression levels of phosphate transporter (PT) genes

**Fig. 5.**
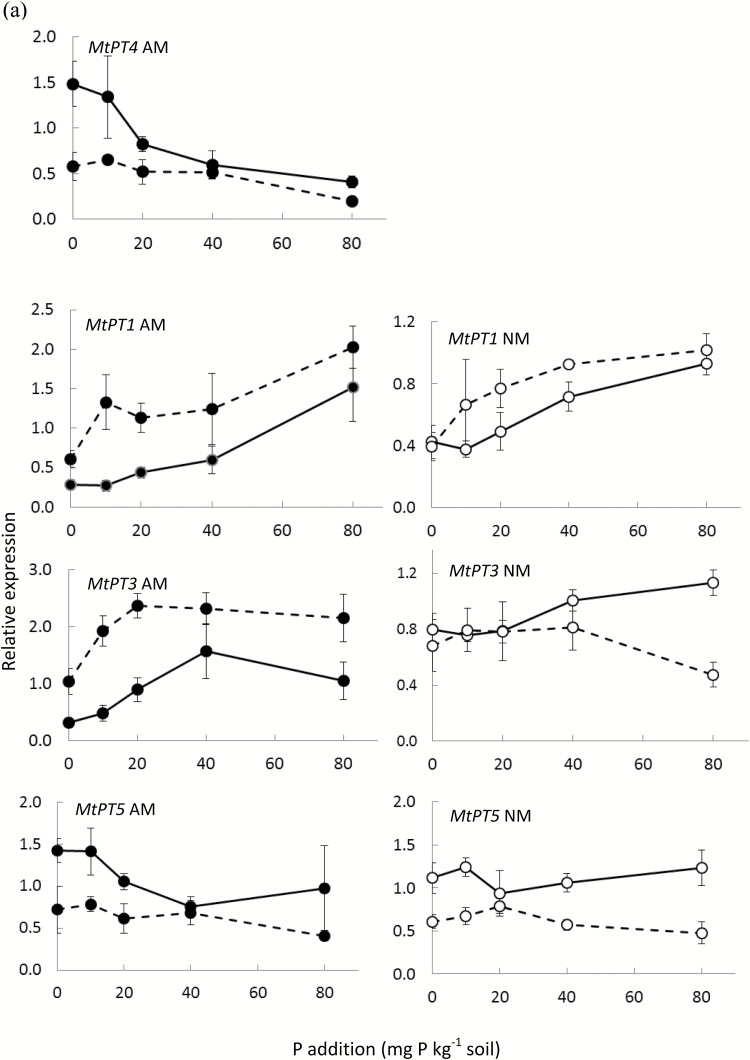

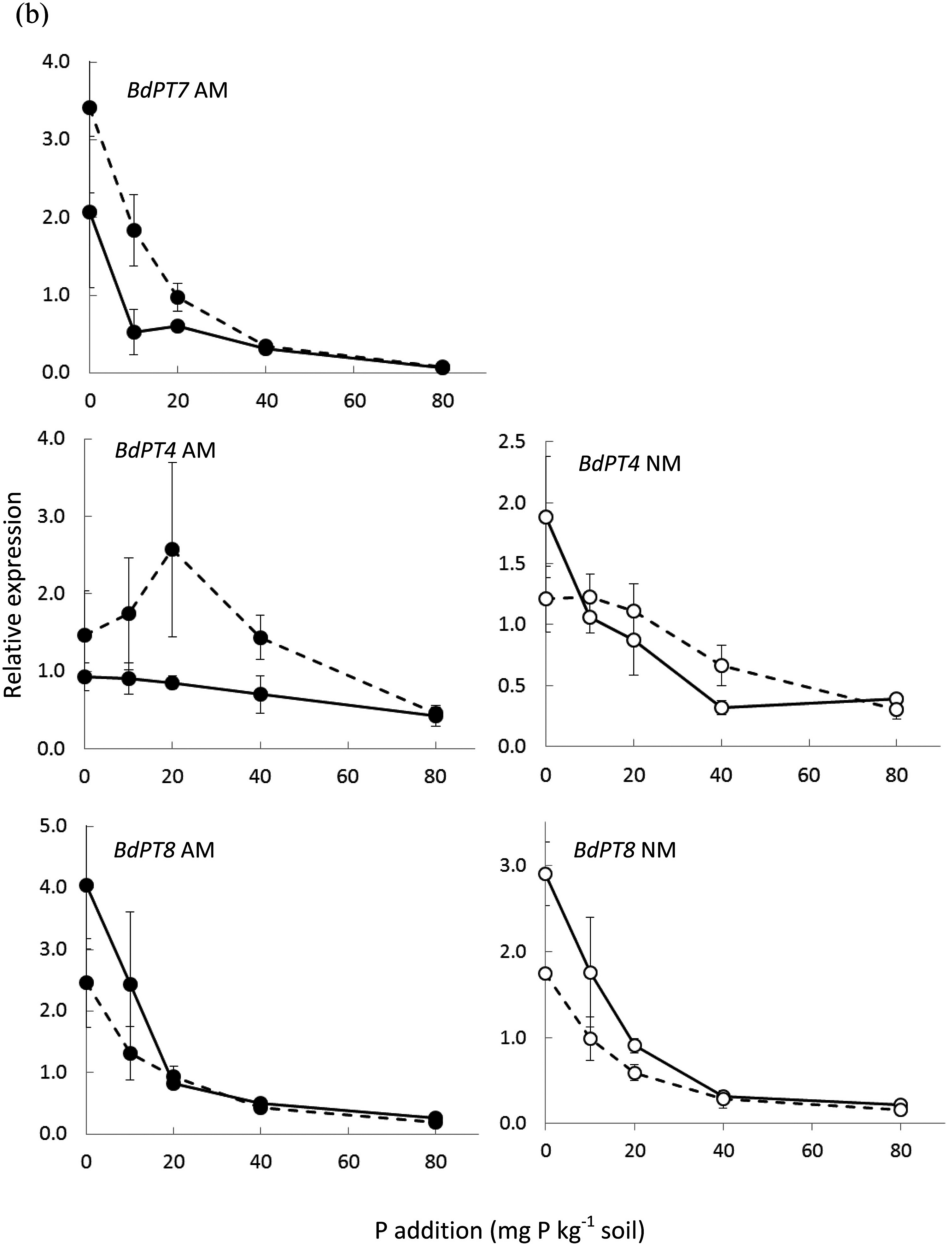
Effects of eCO_2_ and soil P additions on expression of phosphate transporter (PT) genes *MtPT1*, *MtPT3*, *MtPT4*, and *MtPT5* in roots of *M. truncatula* (a) and *BdPT4*, *BdPT7*, and *BdPT8* in roots of *B. distachyon* (b). *MtPT4* and *BdPT7* are induced by AM colonization. Expression levels at aCO_2_ (solid lines) and eCO_2_ (dashed lines) and at different P additions can be compared only within each of the 12 panels. AM and NM plants are represented by filled and open symbols, respectively. Data points are means ±SEM with *n*=3.

Expression analyses of PT genes were performed by RT-qPCR to evaluate the measured contribution of the two P uptake pathways (direct and mycorrhizal) through roots, using ^33^P transfer against expression patterns of genes involved in these pathways. The effects of eCO_2_ on gene expression were analyzed in roots of AM plants and of NM plants separately. Elevated CO_2_ and increasing soil P concentration both decreased the expression of the AM-induced PT gene *MtPT4* in *M. truncatula* (Fig. 5, Table 1). For the direct-pathway PT genes (*MtPT1*, *MtPT3*, and *MtPT5*), the effect of eCO_2_ varied: the expression of *MtPT1* increased and *MtPT5* decreased at eCO_2_ in both AM and NM plants while *MtPT3* expression increased and decreased in AM and NM plants, respectively. The addition of P to the soil decreased the expression of *MtPT1* in both AM and NM plants and of *MtPT3* in AM plants only. Expression of *MtPT5* was not affected by soil P level.

In *B. distachyon*, eCO_2_ had no significant effect on the expression of the AM-induced PT gene *BdPT7*, but its expression decreased with increasing soil P level, as in *M. truncatula* (Fig. 5, Table 1). Direct-pathway PT genes have not yet been characterized in *B. distachyon*. However, phylogenetic studies (Signe S. Clausen, unpublished data; Hong *et al.*, 2012) and expression studies at high and low soil P (Signe S. Clausen, unpublished data) suggest that *BdPT4* and *BdPT8* are active in the direct P uptake pathway. The expression of both genes was slightly increased by eCO_2_ (significant for *BdPT4* in AM plants only and for *BdPT8* in NM plants only). Furthermore, their expression declined with increasing P addition (Fig. 5, Table 1). Trends of declining expression in response to AM colonization were observed for the same two genes in plants grown at the three lowest P levels, but the reductions were not statistically significant (data not shown).

## Discussion

The present work is considerably more comprehensive than previous studies and hence is novel as it exposes plant species of two functional types (a pasture legume and a grass) to factorial combinations of eCO_2_, AM fungal inoculation, and a range of P fertilizer additions under controlled conditions. Our comprehensive physiological measurements (including AM P uptake using ^33^P) and gene expression data have not previously been combined to address our hypotheses. If short-term studies such as this one could be extrapolated to longer-term growth, such a multifactor approach would facilitate the ability to predict effects of future climates on crop nutrition and growth (Cavagnaro *et al.*, 2011). In accordance with our first hypothesis, responses to eCO_2_ under soil P limitation were high in the grass, *B. distachyon*, with a root system that allowed efficient P uptake, but low in the legume, *M. truncatula*, with a less efficient root system. The expected positive relationship between eCO_2_ stimulation of plant growth and increasing P supply (second hypothesis) was confirmed for both species. Furthermore, AM symbiotic functioning was not influenced by eCO_2_ (confirming our third hypothesis), at least in terms of % root length colonized and symbiosis-mediated plant P uptake. In general terms, at the time of experimental harvest, under eCO_2_ less P fertilizer was required to produce the same amount of shoot dry matter as obtained in plants grown at aCO_2_ at non-limiting P fertilizer supplies.

### CO_2_ fertilizer effects on plant growth: interaction with soil P level and mycorrhiza

Our finding that the CO_2_ effect was enhanced at increasing soil P availability in *B. distachyon* and *M. truncatula* supports previous observations that plant growth responses to eCO_2_ are lower under nutrient-limited conditions (Poorter and Perez-Soba, 2001; Nord *et al.*, 2015; Pandey *et al.*, 2015*b*), particularly in relation to N supply, which has been more extensively researched than P. The larger growth response to eCO_2_ in *B. distachyon* than in *M. truncatula* accords with free-air CO_2_ enrichment (FACE) studies showing a greater stimulation of photosynthetic C uptake in grasses than in legumes (summarized in Leakey *et al.*, 2009). This would be expected from the long-established, but sometimes overlooked, ‘law of the minimum’ (Liebig, 1843) and more general principles of plant growth-limiting factors (Blackman, 1905). Put simply, where growth is strongly limited by soil P supply, it would not be increased by eCO_2_ unless there is a physiological interaction whereby higher C supply directly increases P uptake or PUE, or both. There was no such interaction in *M. truncatula* at the lowest P levels (Fig. 1, Supplementary Fig. S1), while at higher, but still growth-limiting soil P levels, eCO_2_ supply increased growth. Possible reasons for such C/P co-limitation are discussed by Pandey *et al.* (2015*b*).

Whereas many legumes are potentially strongly P limited under eCO_2_ (e.g. Edwards *et al.*, 2005; Rogers *et al.*, 2009), P fertilization does not always increase the eCO_2_ response in grasses (Grunzweig and Körner, 2003) and this variation appears to relate to differences in root production between the two plant groups (*B. distachyon* had higher root length at low P). The limited growth response to eCO_2_ in the highly P-responsive *M. truncatula* exposed to low soil P accords with previous findings in chickpea, field pea, barrel medic, and soybean (Sa and Israel, 1998; Jin *et al.*, 2012; Lam *et al.*, 2012; Singh *et al.*, 2014). In contrast, the ~50% growth response to eCO_2_ over the full soil P range in *B. distachyon* is very much in line with a predicted 46% increase in C gain in C3 plants at the atmospheric concentrations of CO_2_ expected for the middle of this century (Leakey *et al.*, 2009). In *B. distachyon*, the lower eCO_2_ response at 80P reflected the observation that plants became C saturated at a lower P level under eCO_2_ than under aCO_2_ conditions.

*Medicago truncatula* displayed the expected AM growth response under P-limiting growth conditions (see for example Smith *et al.*, 2004; Li *et al.*, 2005; Konvalinkova *et al.*, 2015) while *B. distachyon* exhibited neutral or negative AM growth responses. Such response patterns are typical for the vegetative growth phase of grasses such as barley (Grace *et al.*, 2009) and wheat (Li *et al.*, 2005; Stonor *et al.*, 2014), suggesting that *B. distachyon* provides a suitable model for (temperate) grasses with regards to AM research (Brkljacic *et al.*, 2011; Hong *et al.*, 2012). The borderline significant AM×CO_2_×P interaction for shoot DW in *B distachyon* (Table 1) reflected a P level-dependent AM response at aCO_2_ but not at eCO_2_. This AM response was negative at aCO_2_ above 20P and its absence at eCO_2_ suggests that any C drain by the AM fungi that might decrease growth at high-P conditions was fully compensated by increased C assimilation at eCO_2_.

The slight enhancement of % AM colonization caused by eCO_2_ in *B. distachyon* and the lack of effect in *M. truncatula* agree with many previous reports (e.g. Rillig *et al.*, 1999; Gavito *et al.*, 2002, 2003; Cavagnaro *et al.*, 2007), although a meta-study has reported an average increase of 21% (Alberton *et al.*, 2005). However, the eCO_2_-elicited increase in root length means that the absolute colonized root length was markedly increased (up to 50% in *B. distachyon*) and thus the absolute growth of the AM fungi must have responded to eCO_2_ at the same rate as root growth, as also suggested by previous studies (Staddon *et al.*, 1998; Alberton *et al.*, 2005).

### Plants at eCO_2_ have unchanged P uptake efficiency but P use efficiency is increased

The observed lack of CO_2_ fertilizer effects on the P uptake capacity per unit root length in both plant species confirms previous reports (Newbery *et al.*, 1995; Jin *et al.*, 2012) and the observed increased P uptake at eCO_2_ may be explained by the increased root growth (Fig. 2), which contributes to shorten the diffusion pathway in the soil for Pi. External hyphae of AM fungi also contribute to shorten the diffusion pathway for Pi and the calculated AM-mediated Pi uptake generally correlates with the length density of AM hyphae in the soil (Supplementary Fig. S3; Jakobsen *et al.*, 1992; Munkvold *et al.*, 2004). The lack of an eCO_2_ effect on the contribution of the AM pathway to total P uptake in *M. truncatula* and *B. distachyon* adds to previous studies with pea (Gavito *et al.*, 2002, 2003), but is novel by finding this lack of interaction to be independent of P level. This suggests that AM development and function was not C-limited at aCO_2_. In this context, it remains unclear why the growth of AM hyphae into the HC and hence their uptake of ^33^P were greatly reduced at the two lower soil P levels in *M. truncatula* plants, but it has been recognized that AM development can be impaired under extreme P limitation (Bolan *et al.*, 1984). The calculated % contribution of the AM P uptake pathway at 0 and 10P was much lower than that expected from the higher biomass and P contents of AM versus NM plants (Figs 1 and 3b). A possible explanation is that hyphal length density in the main pots (not measured) was higher than in the HCs, perhaps due to slow growth into HCs at low P, that would lead to under-estimation of the size of AM P uptake as derived from the equation used. However, the % contributions of AM and direct uptake calculated for *B. distachyon* did not show low % contribution of AM P uptake at low soil P (Fig. 4).

Our observation that eCO_2_ reduced shoot P concentrations in *M. truncatula* (Fig. 3a) agrees with reports that tissue P and N concentrations are often lower at elevated than at aCO_2_ due to greater plant biomass and carbohydrate accumulation (Treseder and Allen, 2000; Jifon *et al.*, 2002). In general, it has been observed that shoot P concentrations are lower or unchanged under eCO_2_, regardless of AM status (Newbery *et al.*, 1995; Syvertsen and Graham, 1999; Gavito *et al.*, 2000, 2003; Jifon *et al.*, 2002; Jin *et al.*, 2012). Phosphorus concentration (hence also phosphorus utilization efficiency) was not affected by AM in *B. distachyon*. This is as expected because this species is P efficient when NM. In both species, the absence of an AM effect on PUE was also revealed by the similar shoot DWs of AM and NM plants at each CO_2_ treatment when comparing plants of identical shoot P content and hence P physiology (Fig. 3b). In contrast, shoot DW was higher at eCO_2_ than at aCO_2_ at identical shoot P contents in *M. truncatula* above 3–4 mg P per plant and in *B. distachyon* over the full range of shoot P contents (Fig. 3b). This suggests that the P fertilizer requirement to produce maximum growth in a P-efficient grass (at least in the short-term as studied here) is smaller at eCO_2_ than at aCO_2_. In the longer term the additional fertilizer needed for full plant development will depend on several factors, including the fact that much of the total plant P is absorbed during early plant growth and the pattern of redistribution of P within the plant, for example to supply developing seed. Effects of eCO_2_ on such factors will need to be determined in future research. In any case, the potentially smaller fertilizer requirements in the grass might dampen the expected need for increased exploitation of the non-renewable P rock reserves under eCO_2_ (Jin *et al.*, 2015).

### Exposure to eCO_2_ modulates the expression of phosphate transporter (PT) genes

In contrast to the absence of eCO_2_ effects on root length-specific P uptake (see above), eCO_2_ influenced the expression of PT genes in roots of *M. truncatula* plants, both in the presence and absence of AM fungi. However, this expression was rather inconsistently induced or suppressed across PT genes and AM treatments. This potentially reflects the high complexity of regulation of PT gene expression, involving P supply, P starvation responses, and AM colonization, and with many shared components interconnected with sugar and phytohormone signalling (see Smith *et al.*, 2011, and references therein). The effects of eCO_2_ have yet to be incorporated into this picture. In *B. distachyon*, the magnitude of eCO_2_ effects on PT gene expression was much lower than for *M. truncatula*. These results contribute to a field where knowledge is limited: eCO_2_ enhanced the expression of transcription factors and PT genes in P-deficient *Arabidopsis thaliana* plants (Niu *et al.*, 2013), but subsequent *in silico* analysis of typical P-responsive genes of *A. thaliana* revealed no significant influence of short-term exposure to eCO_2_ on PT gene expression (Pandey *et al.*, 2015*b*). The present work on CO_2_×P level interactions in AM species adds to some studies of interactive effects of eCO_2_ and abiotic stress, e.g. drought (Allen *et al.*, 2011; Sicher and Barnaby, 2012; Zinta *et al.*, 2014). The lack of correlation between PT gene expression and root length-specific P uptake is in accordance with previous studies (Grace *et al.*, 2009; Grønlund *et al.*, 2013; Facelli *et al.*, 2014; Watts-Williams *et al.*, 2015). This lack of correlation might be caused by the multiple levels of post-translational regulation of PT genes, as reported in *A. thaliana* (Bayle *et al.*, 2011; Chen *et al.*, 2011, 2015), or by the fact that the amount of transporter protein is not the factor that limits P uptake by either the direct or AM pathways. Alternative limiting factors might well be the concentration of P in the soil solution at the uptake sites or the surface area available for uptake.

While eCO_2_×P effects were not observed in either of the plant species, the PT genes were in most cases regulated by P level, as expected from earlier work in *M. truncatula* (Chiou *et al.*, 2001; Grunwald *et al.*, 2009; Christophersen *et al.*, 2012) and *B. distachyon* (S.S. Clausen, E. Hammer and M. Grønlund, unpublished data). The slightly increased expression of *MtPT1* (NM and AM roots) and *MtPT3* (AM roots) under high P conditions is in contrast to reports of expression of direct- uptake PT genes in *M. truncatula* being suppressed by high Pi in systems with continuous liquid nutrient supplies (Chiou *et al.*, 2001; Grunwald *et al.*, 2009), but is in accordance with results from experiments with more realistic soil-based growth media (Christophersen *et al.*, 2012; Watts-Williams *et al.*, 2015). The expression of the AM-induced *MtPT4* decreased with increasing soil P, as previously reported for *M. truncatula* (Christophersen *et al.*, 2012) and for homologues in tomato (*Solanum lycopersicum* L.) and petunia (*Petunia hybrida* hort. ex E. Vilm.) (Nagy *et al.*, 2009; Breuillin *et al.*, 2010). This negative effect of high soil P availability on expression of *MtPT4* occurred despite a high level of colonization by AM fungi at 80P. This correlates well with the percentage of P uptake via the AM pathway dropping to background levels above 40P, where *MtPT4* expression was also low, as was observed in tomato (Nagy *et al.*, 2009). The low relative contribution by the AM pathway at the lowest P level was associated with reduced growth of the root-external hyphae and was probably caused by P deficiency in the strongly AM-dependent legume. This P-dependent reduction of AM-derived P uptake was not influenced by CO_2_ levels. Expression of PT genes was more strongly suppressed by P in *B. distachyon* than in *M. truncatula*. The clear P-induced repression of the three *B. distachyon* PT genes concurs with reports for other plant species, including barley (Huang *et al.*, 2011) and wheat (Liu *et al.*, 2013). However, the expression *BdPT4* in AM roots was overall low and not significantly affected by P.

## Conclusions

It might be argued that the short-time nature of our experiments reduce their relevance for crops. However, the species we chose are both annuals with short life-cycles of between 8 and 12 weeks, with harvest at 5 weeks representing about half this time, during which they would have taken up more than half of their final total P. Furthermore, *M. truncatula* is a pasture legume so short-term vegetative biomass represents the ‘crop’. Both species would normally be mycorrhizal in field situations. We therefore consider that these model plants provide a good starting point for analysis of effects of eCO_2_ in the contexts of AM colonization and P nutrition.

As already noted, the length of the growth period in this study was limited by the half-life of the ^33^P used to track P uptake via the AM pathway, and it is premature to extrapolate to later harvests and effects on yields of biomass or seed, particularly for crops such as wheat with much longer life-cycles. Obtaining data for such later growth stages will require modifications of the compartmented pot system to allow addition of ^33^P to HCs at different times during plant development. Nevertheless, the results presented here show that no consistent effect of eCO_2_ in different plant species can be expected over a range of soil P levels, especially where growth is limited in low-P soils, as in nature. With higher soil P (more agricultural conditions), and hence lower P limitation, eCO_2_ produces higher growth, but there are differences among species, as there are with responses to AM colonization. Therefore, it is not surprising that meta-analyses have revealed large consistent variations in responses of plant growth to eCO_2_ (Treseder, 2004; Parmesan and Hanley, 2015).

The increased P use efficiency (PUE) at eCO_2_ indicates that there may be no immediate requirement to increase agricultural P inputs in order to capture the expected CO_2_ fertilizer effect. We found little effect of eCO_2_ on % root length colonized by AM fungi or on AM function in terms of calculated P uptake in either of the plant species examined, indicating that the plants maintained proportional C supply to the roots and to the AM fungi regardless of CO_2_ fertilization. The primary role of AM symbiosis under future growth conditions will remain to ensure an adequate P uptake, but there may be no change in the relative impact of the AM P uptake pathway on total P uptake. Effects on uptake of other plant nutrients, especially soil nitrogen, remain unexplored. However, maintaining a balanced C supply for nutrient uptake directly through the root epidermis and AM fungi seems important and is likely to involve a range of signaling mechanisms. Extrapolation to long-term effects on plant growth and yield is even more challenging, because gradual changes in eCO_2_ levels are likely to affect the make-up of AM fungal communities in soil, and AM fungal taxa show differences in C–P trade balance with their host plants (Cotton *et al.*, 2015). However, the role of AM symbiosis in agriculture may find itself gaining more recognition as it becomes one of maintaining general plant fitness, e.g. by improving tolerance to drought and to damaging effects of some pathogens that might be altered under future climates.

## Supplementary data

Supplementary data are available at *JXB* online.

Table S1. Primers for RT-qPCR on *BdPT4*, *BdPT8*, and *BdPT7*.

Figure S1. Root dry weights of *M. truncatula* and *B. distachyon*.

Figure S2. Shoot P content of *M. truncatula* and *B. distachyon*.

Figure S3. ^33^P uptake vs. hyphal length density in *M. truncatula*.

Supplementary Data
