# Supplementary material for: Plant growth responses to elevated atmospheric CO2 are increased by phosphorus sufficiency but not by arbuscular mycorrhizas
Source: J Exp Bot. 2016 Oct 17;67(21):6173–86. doi: 10.1093/jxb/erw383 (PMC5100028; doi:10.1093/jxb/erw383)
Supplement: Supplementary Data [file supp_67_21_6173__index.html]

Plant growth responses to elevated atmospheric CO2 are increased by phosphorus sufficiency but not by arbuscular mycorrhizas — Plant growth responses to elevated atmospheric CO2 are increased by phosphorus sufficiency but not by arbuscular mycorrhizas — Supplementary Data 

# Plant growth responses to elevated atmospheric CO2 are increased by phosphorus sufficiency but not by arbuscular mycorrhizas

## Supplementary Data

Data files

- supplementary\_table\_S1\_figures\_S1\_S3.pdf - Supplementary Data
