# Supplementary material for: Plant growth responses to elevated atmospheric CO2 are increased by phosphorus sufficiency but not by arbuscular mycorrhizas
Source: J Exp Bot. 2016 Oct 17;67(21):6173–86. doi: 10.1093/jxb/erw383 (PMC5100028; doi:10.1093/jxb/erw383)
Supplement: Supplementary Data [file supp_erw383_supplementary_table_S1_figures_S1_S3.pdf]

Supplementary data

Table SI. Primers for RT-qPCR on BdPT4, BdPT8 and BdPT7

Target gene	Bradi number	Primer name	Sequence	Annealing temperature(°C) used	Expected product size (bp)
BdPT4	1g75020	BdPT4-F	5'-GCATCCGGGCCGCGCTCTTCG-3'	60	146
		BdPT4-R	5'-GACCGGGGCAACGGCGTCCTC-3'		
BdPT8	3g12590	BdPT8-F	5'-GTATAAGGCCGGGATCGGGGTCAG-3'	60	141
		BdPT8-R	5'-GTGACCGGCTGGTTCTCGCCG-3'		
BdPT7	2g45520	BdPT7-F	5'-CGACGTCAAGAACATCACCAGGGCG-3'	60	144
		BdPT7-R	5'-GCACCATTGCCAGTATTGCCGTCCTC-3'		

Based on the sequences retrieved from the Gramene database (http://www.gramene.org/) using the accession numbers BRADI1G75020 (BdPT4) and BRADI3G12590 (BdPT8) and BRADI2G45520 (BdPT7). All primers were designed using the primer design tool available from the CLC Main Workbench.

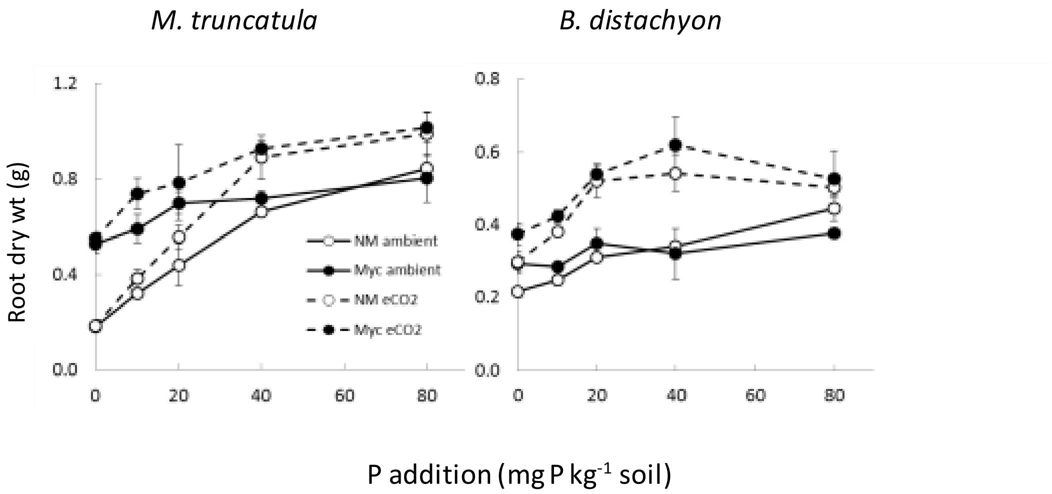

Fig. S1. Root dry weight of *M. truncatula* and *B. distachyon* grown at aCO_2 (solid lines) and eCO_2 (dashed lines), in the presence or absence of arbuscular mycorrhiza (AM or NM: filled or open symbols) and at different soil P levels. Data points are means ± SEM for n=3.

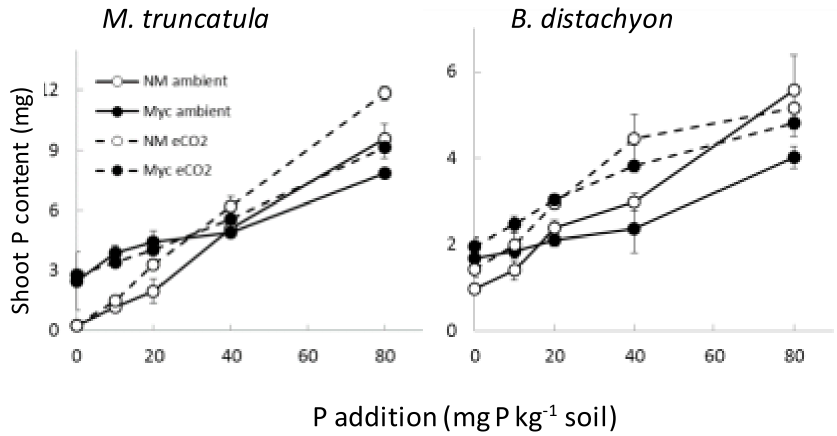

Fig. S2. Shoot P content of *M. truncatula* and *B. distachyon* grown at aCO_2 (solid lines) and eCO_2 (dashed lines), in the presence or absence of arbuscular mycorrhiza (AM or NM: filled or open symbols) and at different soil P levels. Data points are means ± SEM for n=3.

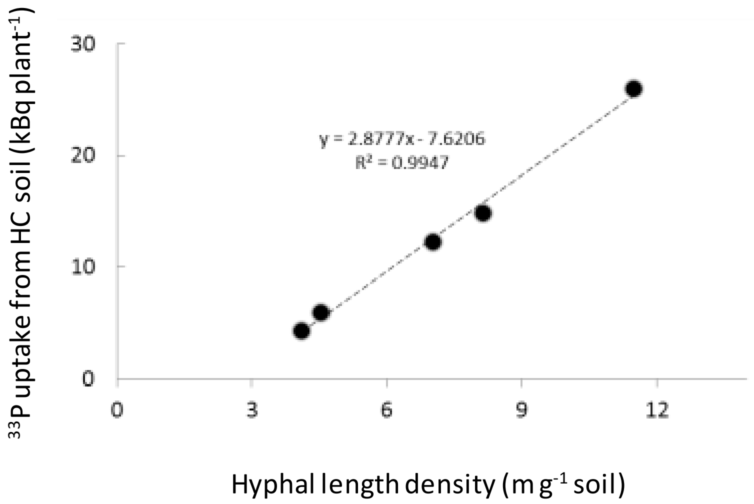

Fig. S3. Correlation between hyphal length density (HLD) in hyphal compartments (HC) and ^{33}P uptake into *M. truncatula* grown at aCO_2. A similar relationship was observed at eCO_2 (data not shown). The equation for the linear relationship was generated in Excel.
